# Proteomic Challenges: Sample Preparation Techniques for Microgram-Quantity Protein Analysis from Biological Samples

**DOI:** 10.3390/ijms16023537

**Published:** 2015-02-05

**Authors:** Peter Feist, Amanda B. Hummon

**Affiliations:** Department of Chemistry and Biochemistry, Integrated Biomedical Sciences Program, and the Harper Cancer Research Institute, 251 Nieuwland Science Hall, University of Notre Dame, Notre Dame, IN 46556, USA; E-Mail: Peter.E.Feist.2@nd.edu

**Keywords:** proteins, mass-spectrometry, mass-limited samples, microgram samples

## Abstract

Proteins regulate many cellular functions and analyzing the presence and abundance of proteins in biological samples are central focuses in proteomics. The discovery and validation of biomarkers, pathways, and drug targets for various diseases can be accomplished using mass spectrometry-based proteomics. However, with mass-limited samples like tumor biopsies, it can be challenging to obtain sufficient amounts of proteins to generate high-quality mass spectrometric data. Techniques developed for macroscale quantities recover sufficient amounts of protein from milligram quantities of starting material, but sample losses become crippling with these techniques when only microgram amounts of material are available. To combat this challenge, proteomicists have developed micro-scale techniques that are compatible with decreased sample size (100 μg or lower) and still enable excellent proteome coverage. Extraction, contaminant removal, protein quantitation, and sample handling techniques for the microgram protein range are reviewed here, with an emphasis on liquid chromatography and bottom-up mass spectrometry-compatible techniques. Also, a range of biological specimens, including mammalian tissues and model cell culture systems, are discussed.

## 1. Introduction

Proteins are essential cellular machinery, performing and enabling tasks within biological systems. The variety of proteins is extensive, and the role they occupy in biology is deep and complex; life depends on proteins. Each step of cellular generation, from replication of genetic material to cell senescence and death, relies on the correct function of several distinct proteins. The precision of cellular machinery can be disrupted, however, resulting in disease. Because much of the machinery essential to cell health and survival remains unknown, studying proteins is of great interest and importance. The field of proteomics is the large-scale study of proteins and the proteome and encompasses many techniques, such as immunoassays [1] and two-dimensional differential gel electrophoresis (2-D DIGE) [2,3]. Another group of methodologies that are growing in popularity for protein discovery and analyses are mass spectrometry-based approaches.

There are two main approaches for mass spectrometry-based proteomics, top–down and bottom–up analyses. Top–down methods analyze whole proteins; bottom–up approaches investigate the peptides from digested proteins. There are unique methods of analysis that each group has developed, but they share in common their mode of analysis. In mass spectrometric analysis, the mass-to-charge ratios (*m*/*z*) of molecular species are determined. By collecting this data, compounds in the sample can be identified by comparing against standard databases of compounds and molecules with known masses. From whole protein analysis in top–down proteomics, to peptide analysis in bottom–up proteomics, each particle measured has an *m*/*z* signature detectable by the mass spectrometer. By pairing mass analyzers and detectors, adding equipment in different configurations, and coupling separations and mass spectrometers together, there are virtually limitless possibilities, functionalities, and speeds of data acquisition for mass spectrometry-based proteomic analysis.

Mass spectrometry-based proteomics has advanced rapidly since the advent of “soft” ionization techniques, namely electrospray ionization (ESI) and matrix-assisted laser desorption ionization (MALDI) [4,5,6] in the late 1980’s. Methods of detection previously used for organic chemicals and other sample identifications were adapted for proteomics. Several combinations of ionization sources and mass analyzers are available commercially, each with merits for specific applications. Time-of-flight (TOF) instruments are often coupled with MALDI instruments [7,8]. ESI-TOF instruments can provide high-speed, continuous measurements without compromising resolution. High-resolution Fourier Transform Ion Cyclotron Resonance (FT-ICR) mass spectrometers are costly, but provide unsurpassed data collection power using both electrospray and MALDI ion sources [9]. The invention and commercial distribution of the Orbitrap mass analyzer by Makarov and Thermo greatly increased the proteomic capabilities of mass spectrometry, combining higher sensitivity and improved mass resolution with lightweight benchtop instruments [10]. The high-field compact Orbitrap, introduced in 2011, provided a high mass resolution instrument at a lower cost than Fourier Transform Ion Cyclotron Resonance (FT-ICR) [11]. The most current Orbitrap instrument, the Orbitrap Fusion, has shown promising results in sensitivity and scan speed [12]. With high mass accuracy and specificity, combined with the selectivity of ion traps and quadrupoles, analysis of complex samples, even in small quantities, can be completed with increasing ease and confidence.

Many mass spectrometry methods are used to analyze proteins. Some methods are discovery-based, where samples are analyzed to determine what proteins are present in the sample. Often a high-resolution mass spectrometer is used for this purpose, as the false-discovery rate of protein identifications from peptides rely on highly accurate mass-to-charge measurements [13]. Some methods are targeted, focusing on single proteins of interest and quantifying them in different samples or sample fractions. Highly selective methods using ion traps and quadrupoles are ideal for targeted analysis [14,15,16]. Proteins and peptides can be fragmented in the mass spectrometer for tandem mass spectrometry in a variety of ways, and those fragments are analyzed for *de novo* peptide sequencing [17,18] or peptide mass fingerprinting [19,20,21,22]. The vast majority of peptide identifications are accomplished with fragmentation followed by protein database searches of the resulting fragments. Electron capture dissociation (ECD) [23], electron transfer dissociation (ETD) [24,25], higher energy collisional dissociation (HCD) [26,27], collision-induced dissociation (CID) [9,28,29], and a host of other fragmentation methods are available [30,31,32], each with recommended applications [33]. Furthermore, mass spectrometry methods are often customized within software.

The mass spectrometer is a critical aspect in proteomic experiments; however, the results obtained from the mass spectrometer are limited by the sample. Regardless of the analysis approach used, a high quality sample is critical for a successful experiment. Proteomic analyses depend on the sample containing proteins to analyze. Sample preparation approaches that are time-consuming, or worse, incur massive sample losses, are intolerable. This review will focus on sample preparation strategies for bottom–up mass spectrometry-based proteomics, with brief focus on obtaining samples for analysis.

## 2. Macroscale *versus* Microscale Techniques

Sample preparation for mass spectrometry-based proteomics has many options available when the sample is not limited. To grasp the need for microproteomics, an understanding of large-scale sample preparation is necessary. By understanding macroscale techniques, proteomicists can adapt their methods to suit microscale techniques. All of the macroscale techniques described here involve quantities of sample in the milligram to 100 microgram range, which do not necessitate as much care and precision as microscale samples, generally defined as samples less than 100 μg. Sample loss in the macroscale is not as pressing, for several reasons. The concentration of the sample is easier to maintain with larger quantities, leading to fewer losses in the processing steps. The overall percentage of lost volumes is lower, because milligram quantities of sample require larger volumes to fully solubilize. Milligram quantities can also be divided more easily while maintaining the majority of protein in the sample, including low-abundance species. Large quantities of protein from complex samples are also generally more diverse in their protein composition [8].

### 2.1. Obtaining a Sample for Proteomic Analysis

In order to obtain protein from a biological specimen, the sample must first be harvested from the organism, culture, or patient. Samples can be obtained by several methods. Traditional dissection from animal species, biopsies, blood draws, and additional methods can deliver adequate protein for analysis. The ethics of obtaining samples are not reviewed here, but guidelines are in place for human, animal, and cell culture procedures [34,35,36].

The proteins in the sample must be made readily accessible via lysis and extraction from the cells in the sample. There are numerous methods for lysis and extraction; lysis buffers and mechanical disruption strategies have been reviewed in the past [37,38]. Most common methods involve a chemical lysis and extraction agent, along with some mechanical stimulus that physically breaks apart the cell, allowing the chemical agent to solubilize the available protein [39]. A list of common detergents, including critical micellar concentration (CMC), can be found in Table 1 [40,41].

**Table 1:** Common detergents for mass spectrometry and cell lysis, organized by type. Adapted from Swiderek *et al.* [41] and Thermo-Pierce documentation.

Detergent Name	Type	Molecular Weight	CMC, mM	Mol. Weight (Micelle)	Suggested Removal
Triton X-100	Nonionic	647	0.24	90,000	TCA/Acetone
NP-40	Nonionic	617	0.29	90,000	Acetone
Tween 20	Nonionic	1228	0.06		Acetone
Tween 80	Nonionic	1310	0.01	76,000	Acetone
Octyl Glucoside	Nonionic	292	23–24	8000	Ethyl acetate
Octyl thioglucoside	Nonionic	308	9		Ethyl Acetate
Big CHAP	Nonionic	878	3–4	8781	Filtration
Deoxycholate	Anionic	415	2–6	2000	Acetone, TCA
Sodium Dodecyl Sulfate	Anionic	288	6–8	17,887	Filtration/FASP
CHAPS	Zwitterionic	615	8–10	6149	Filtration
CHAPSO	Zwitterionic	631	8–10	7000	Filtration

Highly efficient lysis and extraction has been achieved using several lysis buffer formulations, with mechanical perturbations ranging from gentle rocking or cell scraping to sonication or French pressing. The choice of lysis buffer and mechanical disruption depends on the protein target of extraction, sample size, and experience in preparation methods.

Lysis buffers can differ in critical micelle concentration (CMC). The CMC is the concentration at which the detergent forms micelles spontaneously, which can affect their efficacy and removal in different environments. Above this point, the detergent forms micelles, and detergent added will move directly into micelles. Higher CMC values are associated with weaker hydrophobic binding to monomers. Thus, higher CMC detergents tend to be more easily removed by buffer exchange and dialysis. Solutions with lower CMC values form micelles more easily, and generally require less detergent to effectively solubilize protein. Another factor that can affect a lysis buffer is the micelle molecular weight (MMW). Lower-weight micelles are more easily removed than higher-weight micelles. Making use of CMC and MMW, one can more easily determine the best course for the experiment. Choosing a lysis buffer depends greatly on these detergent factors. Most of the detergents listed are incompatible with downstream mass spectrometry analysis, and must be removed. There is no absolute “best way” to lyse a sample, as illustrated by the number of lysis and extraction protocols that exist in the literature; however, advantage lies with speed, sample retention, and cleanliness of preparation.

Lysis, extraction, and denaturation of protein can occur in the same step with certain procedures, such as with sodium dodecyl sulfate SDS while boiling and agitating the sample. Gutstein, *et al.* published an excellent review on microproteomic techniques describing methods for harvesting and lysis [8]. Bodzon-Kulakowska [42], Visser [43], and Hilbrig [44] published comprehensive reviews on sample preparation techniques with methods of cell disruption; Bodzon-Kulakowska has the most recent, large-scale review containing detailed analyses on the extraction of protein and subsequent removal of nearly all types of contaminants [42]. Visser includes classification of different techniques into “hard” conditions, which differ significantly from physiological conditions, and “soft” lysis, which are often used when the biological activity of the analyte needs preservation [43]. Hilbrig focuses on affinity precipitation of proteins [44].

Macroscale proteomic lysis techniques can efficiently extract protein from large amounts of sample. The issue with broadly applying a certain technique to a microscale sample is the removal of detergents and contaminants present in the lysate, which interfere with later steps. Catastrophic losses can occur from indiscriminate application of lysis techniques without proper planning and care. Microproteomic techniques focus on the efficient lysis of sample and removal of contaminants while retaining maximum sample. For example, the French Press has a large surface area where proteins can adsorb and be lost. This mode of lysis is not ideal with microgram quantities of sample. Instead, sonication in microcentrifuge tubes is be a better choice, so as to efficiently lyse cells, maximize sample concentration of protein, and thus minimize loss to surfaces.

### 2.2. Contaminant Removal

Once protein is extracted, removal of contaminants and detergents is necessary. Some detergents will interfere with enzymatic digestion, and most will interfere with reverse-phase separations and mass spectrometry, sometimes damaging instruments and irreversibly ruining columns [45]. Removal of unwanted cellular material, such as lipids and genomic DNA, prevents signal suppression, chromatographic interference, and presents a much cleaner, clearer spectrum from which to obtain protein identification data. One common approach for contaminant removal is precipitation. The uses of precipitations vary based on three main factors: The detergent or contaminants for removal; whether the proteins must be kept in a native or denatured state; and the post-processing analysis. Here, the focus is on bottom–up mass spectrometry-based proteomics; thus, detergent removal is a must, and the denaturation of the protein has little to no bearing. The post-processing analysis is singular, but there are numerous added steps to consider. Some common detergents can be removed using acetone precipitation, the classic precipitation technique in many proteomics and biochemical procedures [46]. Others can be removed using trichloroacetic acid precipitation [47,48,49]; chloroform-methanol mixture [50] or ethyl acetate [51,52] can remove contaminants with high yield, and most interferents can be eliminated using molecular weight cutoff filters to capture the protein in the sample. There are various other precipitation techniques that have been developed and compared for recovery and post-precipitation analysis in gels or for in-solution digestion (Table 2) [53,54,55]. TCA, chloroform-methanol, ethyl acetate, and acetone precipitation have similar efficiency for a wide variety of samples; chloroform-methanol has been found to work better for membrane proteins [49,56], while acetone precipitation sequesters mostly water-soluble proteins.

**Table 2:** Approaches for contaminant and detergent removal in proteomic samples.

Approach	Description	Reference
“Salting out”	Precipitation uses saturation of salt to precipitate protein from solution. Most commonly an ammonium sulfate precipitation, but also uses sodium sulfate.	[57,58,59]
Ultrafiltration	Centrifugation at high speed using molecular weight cutoff filter to remove contaminants; prominent in Filter-Aided Sample Preparation (FASP).	[57,60]
Polyethyleneimine (PEI)	Cationic polymer precipitates nucleic acids in 1 M NaCl, leaving proteins in the supernatant. PEI must be removed before further analysis.	[57,58,61,62]
Isoelectric Point (PI)	The pH of solution is adjusted with mineral acid to the isoelectric point of most proteins (pH 4–6). Neutral proteins will aggregate and precipitate.	[57,63,64]
Thermal	Cell extracts are denatured using heat; denatured proteins aggregate and precipitate, but stability is enhanced.	[57,65,66]
Nonionic polymer Polyethylene glycol (PEG)	Concentration of PEG unique to the protein mixture is added. Proteins precipitate based on an excluded volume principle. Centrifugation pellets the precipitated protein. PEG must be removed before mass spectrometry analysis.	[57,67,68,69]

**Figure 1:**
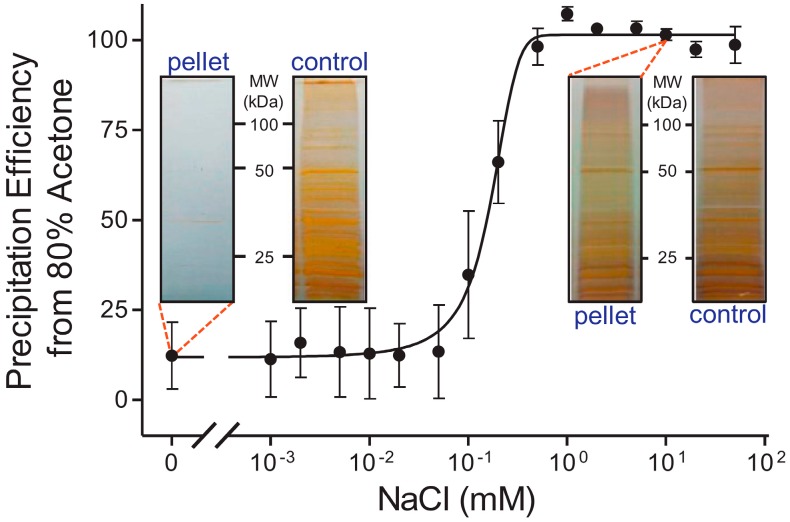
Acetone precipitation efficiency varies with salt concentration. The protein yield from an acetone precipitation in a complex mixture correlates with protein concentration, dielectric strength of the solution, and intrinsic protein charge. The overall model proposed is an ion-pairing model for organic solvents. With this protocol, efficiencies of 100% can be achieved for acetone precipitation. Figure adapted from Crowell, *et al.* (2013) [70].

Acetone precipitation is simple to perform. The standard procedure for acetone precipitation involves the addition of cold (0 to −20 °C) acetone to aqueous sample mixtures to a composition of 80%. Crowell, *et al.* examined the varying reports for acetone precipitation efficiency [70], which ranged from 50% to 100% in the literature [71,72,73,74,75]. After observing that removal of SDS from a protein solution of bovine serum albumin (BSA) caused a precipitous drop in efficiency, despite increasing concentration of BSA, acetone, and non-ionic detergent, they optimized conditions to improve yields from acetone precipitation (Figure 1). They found that the initial concentration of protein obtained during cell lysis is a major factor, as well as the percentage of acetone used and the ionic strength of the pre-precipitation solution. The ionic strength correlated with protein charge and dielectric conditions in the protein sample. Ideal conditions for near-quantitative to quantitative yield of protein were established to include addition of 80% acetone to nearly any protein mixture containing 1 to 100 mM of NaCl or similar salt. The salt concentration necessary for complex mixtures can vary widely, requiring some optimization for particular cell lysates.

Another mature precipitation technique is the methanol-chloroform precipitation. Wessel and Flugge introduced this efficient method of precipitation and concentration for dilute samples, especially those containing membrane proteins. They describe the procedure using a 4:1:3 ratio of methanol:chloroform:water, with an additional three volumes of methanol added to pellet the protein. Proteins that are only slightly soluble in methanol-chloroform collect on the water-organic interface, and are then pelleted by the additional methanol and centrifugation. The efficiency of this procedure approaches 100% for a variety of protein concentrations and detergent solutions. Methanol-chloroform precipitation has uses for bottom–up and top–down proteomics [58]. Ideally, precipitation would be avoided in microproteomics, because of the possibility of total sample loss. However, several factors discussed above that govern precipitation efficiency can be altered to produce successful precipitations, even in the microscale. These manipulations, especially as they relate to protein concentration, will be discussed in detail later in this manuscript.

Dissolving precipitated samples can be a challenge; as the protein precipitate is usually compacted into a pellet by centrifugation steps and the pellet is often dried to some extent. Exposure of the whole pellet to the dissolution buffer is necessary. Part of the precipitate can easily be left behind if thorough examination of the solution and container is not performed [76,77]. Strategies for fully suspending the pellet in any lysis buffer of choice include vigorous vortexing [58,59,78], sonication [58,79], shaking, and even two-step, on-pellet trypsin digestion [80]. In microscale, it is best to avoid vigorous agitation, because adsorption and loss occurs with increased exposure to surfaces; sonication avoids much of the splash-up that vortexing or shaking involves, but efficiently exposes the pellet to buffer.

### 2.3. Digestion Strategies

In-solution and in-gel digestion are two well-used approaches to prepare a bottom–up proteomic sample. In-solution digestion is the one of simplest and most powerful of the macroscale techniques in shotgun proteomics to perform. In-solution digestion involves denaturing, reducing, alkylating, and digesting the protein sample in the liquid phase, as opposed to in a gel or on a filter. In-solution digestion is extremely common and has been used with a variety of samples [81,82,83,84]. In-solution digestion can be performed using single-tube approaches, eliminating much of the sample loss that occurs during solution transfer between different vessels. Generally in-solution digests are fractionated after digestion, but fractionation can be performed previous to digestion using different forms of chromatography, including, but not limited to, strong and weak ion exchange, reverse-phase, and size exclusion chromatography.

**Figure 2:**
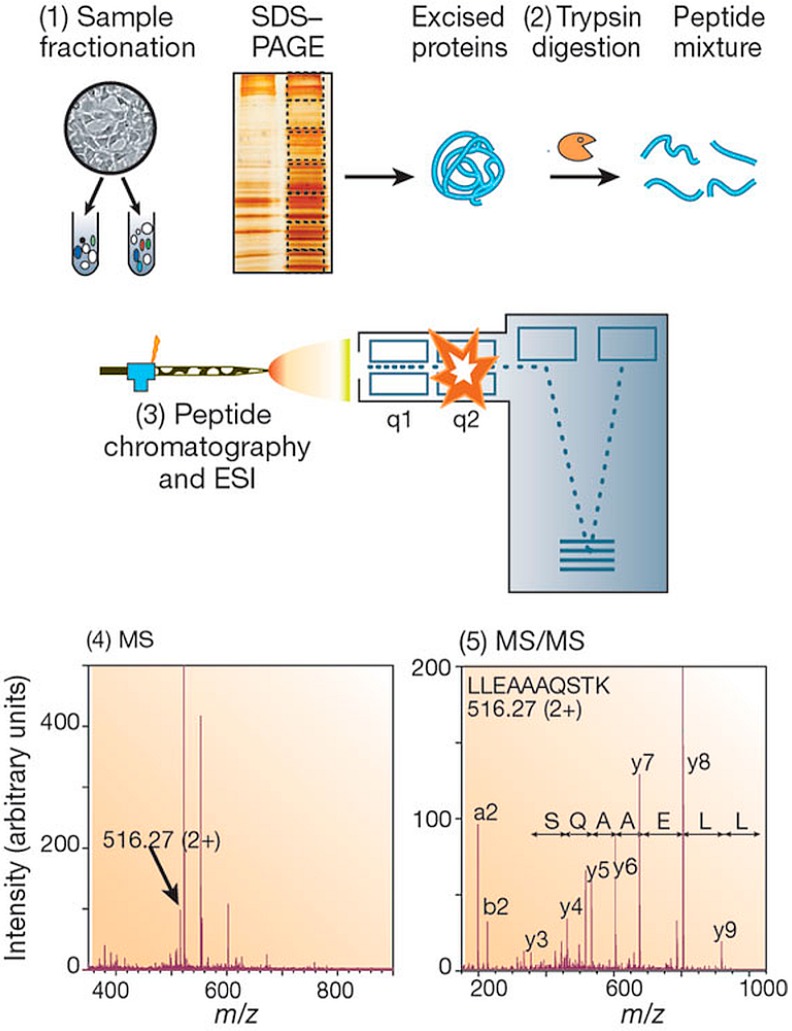
Typical workflow for gel-based mass spectrometry analysis. The gel is used to separate whole protein in one or two dimensions. After destaining, the proteins are excised from the gel and subjected to enzymatic proteolysis. Peptides can then be analyzed via mass spectrometry. Figure adapted from Aebersold *et al.* (2003) [9].

Gel-based mass spectrometry analysis is widely used [85,86,87] as a first method of separation prior to LC–MS/MS analysis. Different methods of gel electrophoresis have been discussed in several reviews by Herbert [88], Lilley [3], Görg [89], Rabilloud [90,91], and others. Before the digestion, separation of the protein is performed using a gel. Basic overviews of gel-based mass spectrometry protocols can be seen in Figure 2 [9]. In one of the most common proteomic sample preparation strategies, a denaturing gel (sodium dodecyl sulfate in a polyacrylamide gel, SDS-PAGE) is used for bottom–up proteomics, as the protein will be cleaved into peptides in later steps [9,92].

The stained gel pieces are then excised from the gel, destained, and the protein within the gel piece is subjected to digestion. Several strategies are commonly used before eventual in-gel digestion, and there are many variations on one- and two-dimensional gel separations. One-dimensional gels are excellent for simple fractionation; proteins often have specific molecular weights, allowing for a semi-targeted approach. Less interference is involved in this approach, as the sample is dramatically simplified, so that only isobaric proteins will exist in the digest. SDS-PAGE is possibly the most widely used proteomic technique today [93], owing to the ability to separate thousands of proteins in a single sample from a complex mixture [94]. SDS-PAGE is also a relatively simple procedure, and the gel provides a good vehicle for the safe storage of proteins for future analyses [93].

Two-dimensional (2D) gels add time to the protocol, but increase the selectivity of the process. 2D gels can use more than one orthogonal property of the protein for separation purposes; for example, isoelectric point (P_I_) and molecular weight. One of the main differences between 1D and 2D gels is the fact that separation by P_I_ requires non-denaturing conditions. This condition means that common solubilization procedures using SDS, as well as use of other charge-coating detergents and running buffers, cannot be used in a first dimension isoelectric point focusing. Sample preparation then requires more thought and care. Rabilloud and Lelong have published a tutorial on 2D gel electrophoresis [95], covering the process, limitations, and advantages of the technique. 2D gels are run with different, orthogonal linear coordinates; the gel axes of the first dimension are rotated 90°, so that the proteins migrate on what was previously the horizontal axis, onto a second gel. 2D gel electrophoresis has been used for the fractionation and identification of many thousands of proteins in milligram sample amounts, as well as in the analysis of laser capture microdissection (LCM) samples [94,96].

In-gel digestion has several advantages over in-solution digestion. Using a gel, samples of greater complexity can be analyzed via mass spectrometry, because fractionation, and thus simplification, of the sample occurs on the gel. In-solution digests do not reduce the complexity of the sample without further pre-fractionation. Rabilloud and Lelong generated a cost-benefit comparison of gels *versus* mass spectrometry time [95]. Normal shotgun analysis on a mass spectrometer has a limited dynamic range and can only handle so much complexity, requiring more injections and mass spectrometry time to identify proteins of interest. Gels, used as a pre-fractionation procedure, can decrease the amount of mass spectrometry time needed to obtain the same or better information. Time on the mass spectrometer is generally more costly than even a 2D gel set-up. Gels are also useful for easing the removal of contaminants from the sample that may interfere with mass spectrometry analysis [97].

In-solution digests have the advantage of simplicity; fewer things can go wrong in the straightforward methods of in-solution digests. In-solution digests require less protein, because sample loss is often more severe for gel-based analysis. Sample recovery for gel-based procedures is estimated to be 70%–80% by Granvogl *et al.* [92]; Speicher *et al.* have a similar estimate [98]; extraction of peptides from a gel is inherently less efficient than an in-solution digestion [99], with estimates of 70%–80% of in-solution digest efficiency [93]. Sample complexity can be compensated using pre- or post-digestion fractionation for in-solution digests. Contaminants can be removed, at the expense of some sample loss. Concentration can be roughly controlled in solution, while the quantitative amount of protein from a gel is more difficult to ascertain; a rough estimate is possible based on staining [100].

The advantages of in-solution digestion and in-gel digestion have been combined in the form of spin-filter aided digestion protocols. Filter-aided digestion was originally developed by Manza, *et al.* [101,102] in 2005, and the acronym FASP (for Filter-Aided Sample Preparation) was coined by the Mann group in 2009 [103]. The process has now been widely derivatized for many mass spectrometry protocols and sample compositions [104,105,106]. As discussed by Wiśniewski and colleagues, the proteins in the sample are trapped in a high-molecular weight cutoff filter. Salts and low-molecular weight compounds flow through the filter and can be discarded. One key feature of FASP is the effective removal of detergents and contaminants from samples. The use of urea buffer enables the removal of nearly all (≥99.9%) of SDS from the sample; this is one of the unique identifiers separating Wiśniewski’s FASP from Manza’s spin-filter protocol [107]. After carboamidomethylation of the proteins, trypsin is added directly to the filter. The filter acts as a reactor for the trypsin digestion. Once on-filter trypsin digestion is accomplished, the peptides can be eluted in whatever volume of buffer necessary, and the filter retains any high-molecular weight interferents. FASP is a single-tube protocol, substituting aqueous- or organic-phase digestion for a solid-phase, reactor-based protocol. Sample losses have been variably reported [101,102,108,109]; however, whether these are technical variations or downfalls in the protocol are unknown. Nevertheless, the FASP protocol has been improved upon in recent years, with evidence of efficient low-microgram sample analysis [110]. In a comparison of bottom–up approaches by Weston *et al.*, FASP outperformed in-solution digestion techniques with a 66.2% identification rate, as opposed to 47.8% for the in-solution digest [111]. In our experience, individual protein bands become difficult to visualize and excise with traditional Coomassie Blue staining, especially with sample amounts less than forty micrograms of complex protein mixture. Silver staining methods have arisen that alleviate this problem [95]. The peptide recovery of in-gel digestion also affects its use in microscale, *versus* the higher recovery efficiency of FASP or in-solution digests.

### 2.4. Fractionation and Separation of Proteomic Mixtures

Various mass spectrometry and fractionation combinations have been developed or refined with the intent of delving deeply into the proteome of organisms and model systems. For the yeast proteome, the multi-dimensional protein identification technology was developed by the Yates research group [112]. Accurate mass tags (AMT) were developed in order to decrease the need for tandem mass spectrometry while providing more sensitive measurements and greater dynamic range [113]. High-performance liquid chromatography (HPLC) is a very common separation technique with a wide variety of stationary phases. With the advent of ultra-performance liquid chromatography (UPLC), chromatographic separations have increased both in resolving power and speed of separation. HPLC and UPLC function on the same principles. UPLC columns generally offer smaller particle sizes, resulting in decreased analyte path length and higher column pressures (10,000 pounds per square inch (PSI) or greater in maximum). Liquid Chromatography is often coupled with electrospray ionization and tandem mass spectrometers for both top–down and bottom–up proteomic studies. LC has been used for a staggering number of analyses [114,115]; LC–MS is a proteomic workhorse [116]. Liquid chromatography is robust, customizable based on the functionality of the stationary particles in the separation column.

For bottom–up proteomic analysis, the most common HPLC/UPLC stationary phase is the C18 reverse-phase column. The reverse-phase column uses the hydrophobicity of peptides for separation, utilizing a gradient from low to high organic-phase solvent. Acidified methanol and acetonitrile are commonly used as organic-phase, also known as “B” or “strong” solvents because of their miscibility with aqueous solutions. Acidified water is most often the “weak” solvent, also known as “A”. Both buffers are acidified with the same acid, generally with formic acid or trifluoroacetic acid (TFA) at 0.1% or 0.01%, respectively. Formic acid is preferred over TFA, as TFA tends to form adducts and suppress signal [117,118]. While reverse-phase columns are very popular, many stationary phases are in use for proteomic work in both one- and two-dimensional separations, online and off-line. A separation strategy known as electrostatic repulsion hydrophilic interaction chromatography (ERLIC) has gained popularity for phosphoproteomic work, using adjustments in pH and volatile salts for gradient separations. As the name suggests, ERLIC uses the charge and hydrophilicity of peptides as a basis for separation. Typically ERLIC begins with a low-organic, high-pH gradient, moving to high organic and low-pH as the separation moves on. In this way, ERLIC elutes peptides in order of increasing hydrophobicity and acidity. ERLIC has proven effective at separating and identifying modified and unmodified proteins [119,120,121,122]. Smaller-diameter columns with lower loading capacities and smaller stationary phase particles offer an advantage in microproteomics. By increasing the local concentration of peptide and decreasing eddy diffusion, sample loading amounts can be minimized and still provide adequate peptide signal; the chromatographic resolution necessary for complex sample separation is not compromised.

## 3. Microproteomics

Sample size and complexity are challenges in mass spectrometry. The sub-field of microproteomics focuses on improved analysis of microgram-quantities of samples through careful sample handling and increasing efficiency of processing. Depending on extraction techniques, tissue type, and cell density, 1 to 100 micrograms of protein can be obtained, processed with microproteomic techniques, and analyzed, with tissue remaining for use in other techniques. Alternatively, laser capture microdissection can be used to obtain samples for microproteomics post-microscopy analysis, providing thousands of cells for diagnostic analysis [94,96,123,124,125]. Complex samples are processed in a different manner than simple protein mixtures. Cells and tissue have a vast range of protein amounts, from 100 or less copies per cell to 10^6^ copies for yeast [126]; it is conceivable that the dynamic range is greater in mammals [127,128]. Complex mixtures require fractionation prior to mass spectrometry analysis, while simple protein mixtures can be separated in line with the mass spectrometer. With micrograms of sample available, fractionation becomes a greater challenge. Successive sample losses can be crippling; dilution of sample in complex mixtures can mean losing low-abundance protein species below signal-to-noise cutoffs. Microproteomics techniques help ensure the maximum amount of sample possible, hopefully allowing for detection and identification of even low-abundance proteins in the sample.

As with macroscale analyses, obtaining sample is the first defining step of microproteomics analysis. Microproteomic techniques often rely on precise excision of tissue as much as preparing the sample with care from start to finish. Laser capture microdissection allows for the precise removal of several thousand cells or less from a fixed, microscopy sample. Other methods for obtaining sample are also viable. Wang *et al.* used flow cytometry to obtain 500 to 5000 cancer cells in their circulating tumor cell simulation [129]. Sun *et al.* used *Xenopus* embryos and ova, measuring roughly 1.2 mm in diameter [130]. Smaller organisms or parts of organisms can be homogenized or dissected to provide microproteomic samples as well. The lower limit on complex proteomic sample sizes has not been firmly established; descriptions of nanogram analyses have been reported [94,123,129].

### 3.1. Sensitivity and Microscale Analysis

Advances in instrumentation benefit the field of microproteomics, lowering the threshold of detection for less abundant proteins in samples, which would normally be lost with less sensitive instrumentation. Although Fourier Transform Ion Cyclotron Resonance (FT-ICR) instruments, which provide unmatched mass resolution and mass accuracy, are powerful tools, they are not ideal for microproteomics. These instruments are expensive and compromise scan speed as mass resolution is increased; the hybrid Orbitrap family is more routinely used [131]. The advent of the Orbitrap mass analyzer provided a sensitive benchtop alternative with speed and selectivity, furthering the utility of microproteomics and decreasing sample size requirements, in some cases down to single cell measurements [9]. Comparisons between the LTQ Orbitrap Velos, a linear ion trap-Orbitrap hybrid mass spectrometer, and the Q-Exactive, a hybrid quadrupole-Orbitrap system, show increases in protein identification and the superiority of each instrument in different modes of fragmentation on a reasonably complex proteomic sample. Sun *et al.* determined that the Q-Exactive outperformed the Orbitrap Velos over a range of loading amounts from 1000 to 1 ng on a Waters NanoAcquity UPLC system. For this system, it is also notable that the CID fragmentation mode was out-performed by HCD fragmentation on the Orbitrap Velos [132].

Increases in sensitivity are not always due to advances in technology. Meyer *et al.* reported an increase in electrospray sensitivity with the addition of low percentages (~5%) of dimethyl sulfoxide (DMSO) to separation buffer systems [133]. Upon investigation with an Orbitrap Elite system, Hahne *et al.* determined that an increase in electrospray efficiency and charge state reduction, due in part to the high proton affinity of DMSO in the gas phase, were major factors. With a charge-state reduction experiment, DMSO strips multiply charged species of protons, leaving more doubly charged peptides for detection, thus increasing signal and decreasing ion injection times [134]. While appealing, there are conflicting views on the use of DMSO in mass spectrometry [133,134,135,136], possibly due to conjugation product that form when DMSO sits for long periods of time, as well as the high boiling point of DMSO itself (189 °C). One adduct, dimethylsulfone, has a very high boiling point (238 °C) and thus does not ionize to gas phase in electrospray ionization. Accumulation of dimethyl sulfone as a jelly-like substance will damage a mass spectrometer or column, so freshly distilled DMSO or no DMSO may be preferable, despite possible gains.

### 3.2. Clean Sample Preparation

Microproteomics focuses on the separation, preparation and analysis of protein samples under 100 micrograms, which are very sensitive to losses in the proteomic workflow. With each transfer and processing step, protein can be lost. Reproducibility of sample preparation is key for reducing and troubleshooting sample loss in microproteomics. The concentration of protein plays a large role in sample loss. More concentrated samples tend to have less catastrophic losses in precipitation, desalting, and resuspension, due to the concentration-dependent adsorption maxima of many proteins. Select proteins in the sample will adsorb less to surfaces, such as beads, at higher concentrations compared to lower concentrations [137,138]. Higher concentration minimizes catastrophic sample loss by surpassing the adsorption maxima of proteins in the sample. Microproteomic techniques help ensure minimal sample loss before analysis, and maximal identification of peptides and protein groups during analysis, all while adhering to the strict purity standards necessary for mass spectrometric analysis. For example, some techniques use detergent-free methods of digestion, which lessens the cleanup steps involved in the protocol [139,140,141]. Acetonitrile, ammonium bicarbonate, and Rapigest^©^ have successfully been used in digestion protocols with microgram quantities of protein. Others have developed removal methods that eliminate the protein from solution without precipitation steps, such as the Filter-Aided Sample Preparation protocol. Although many microproteomicists prefer not to precipitate low quantities of samples due to risk of total loss, precipitation remains a viable and effective microproteomics technique.

Because precipitation does not provide 100% recovery in most cases, two main types of precipitations are favored for their ease of use and high recovery: Acetone precipitation and trichloroacetic acid (TCA) precipitation in deoxycholate. Acetone precipitation is a concentration-based procedure; higher concentrations of protein in the original solution result in higher recovery [142]. In an acetone precipitation, efficiency ranges from 50% to 100% recovery, with dependency on ionic strength of the solution as well as initial protein concentration. If acetone precipitation is to be used for microproteomics, it is ideal with lysis reagents, such as Nonidet P-40 (NP-40), which obtain good protein yields during extraction [40,129]. Even nanoscale analyses can be performed using acetone precipitation. Wang, *et al.* analyzed the proteome of 500 to 5000 cancer cells using NP-40 lysis buffer and acetone precipitation [129]. Using a commercial reverse-phase liquid chromatography separation, electrospray ionization, and quadrupole-time-of-flight (Q-TOF) mass spectrometer, an average of 619 ± 59 proteins were identified from 1.4 μg peptides obtained from 5000 cells; an average of 167 ± 21 proteins were identified from an estimated 140 ng peptides obtained from 500 cells. The interference from NP-40’s polyethylene glycol units was not observed. Although protein losses occurred during the precipitation, significant numbers of proteins were identified from even the smallest sample amount. With the advances in sensitivity and throughput from newer mass spectrometers, these numbers could improve. Acetone precipitation also works relatively well for SDS removal, with about 100-fold removal [143]. Care must be taken, however, to remove enough SDS to prevent problems with chromatography, as small amounts of SDS can interfere chromatographically with reverse-phase separations [144]. This can be especially troublesome with low-quantity samples.

Quantitative protein precipitation using TCA is specific to deoxycholate and certain other detergents in solution; TCA can be used alone for precipitation, but the pellet is not readily dissolved, causing sample losses [59,145]. In a typical TCA-deoxycholate (DOC) precipitation, the protein solution is mixed with a dilute sodium deoxycholate buffer. The protein is intercalated by deoxycholate and then precipitated from solution with TCA. Acidification of the deoxycholate can cause a change in solubility [49,146], causing aggregation and precipitation. The deoxycholate can then be preferentially removed using acetone, leaving near-pure protein behind [47,48]. TCA-DOC precipitation is remarkably efficient, even in dilute samples, with recovery values from 90% to 100% of total protein [48].

Both the TCA-DOC and acetone precipitation methods have been adapted to the microscale and can recover the majority of protein input. Acetone precipitation is inherently more difficult to perform in the microscale compared to the macroscale, due to the dependence of its efficiency on protein concentration [70]. In either case, the protein is denatured and also separated from materials that interfere with liquid chromatography-mass spectrometry analysis, such as lipids, cellular debris, and non-compatible detergents. The pellet left behind can be dissolved in a buffer of choice. Care must be taken in dissolving precipitated samples, particularly when using TCA, as these pellets can be extremely difficult to re-suspend if over-dried, or if the proteins have limited solubility in the buffer of choice. Often dissolution is performed in a denaturing buffer or chaotropic agent compatible with mass spectrometry, or an agent easily diluted in the sample for the prevention of interference. An 8 M urea or 6 M urea with 2 M thiourea mixture, Rapigest^©^, ammonium bicarbonate, and 80% acetonitrile are common buffers for resuspension. Usually resuspension of a microproteomic sample is performed in small volumes, to keep protein concentration high and prevent losses, so resuspension techniques such as shaking or vortexing are discouraged in plastic tubes. The exposure of protein to the plastic walls may result in irreversible loss. Thus, sonication or on-pellet digestion are better approaches for dissolving precipitation pellets.

Filter-aided sample preparation (FASP) has also been adapted to microscale analysis. Sample preparation is similar to the macroscale techniques described. FASP is considered efficient for a wide range of proteins and preparations, and shows great efficacy for removal of detergents and contaminants without sample losses. FASP has been used for small numbers of cells (3000 and under) from formalin-fixed paraffin-embedded samples with LCM. Wisenewski *et al.* reported 2055 protein identifications from 3000 cells, containing nanograms worth of protein, with no interference from formalin or paraffin [110]. Protein can also be concentrated and desalted in few steps. FASP can be combined with other methods, such as with isobaric tags for relative and absolute quantitation (iTRAQ) [147], a multiplexed approach to protein quantitation, and antibody affinity selection [148].

**Figure 3:**
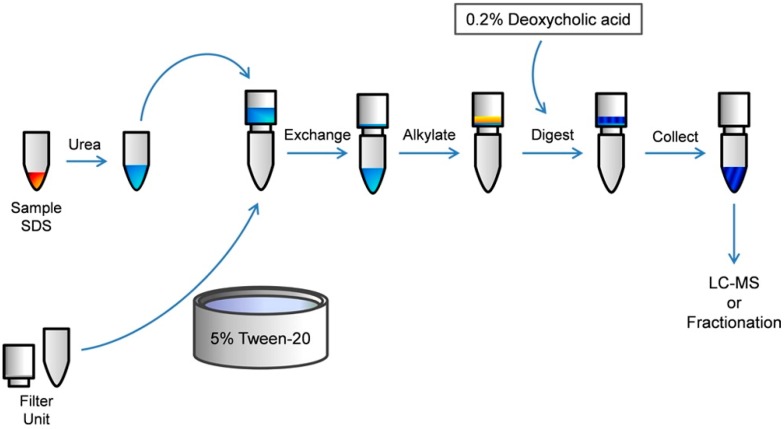
Enhanced filter-aided sample preparation (FASP) workflow. Samples are prepared in 4% sodium dodecyl sulfate (SDS) and diluted in 8 M urea to dissociate SDS from the proteins. Filter units are passivated overnight with 5% Tween-20, followed by thorough washing in MS-grade water. Diluted samples are applied to the filter units for buffer exchanges, eliminating contaminants. Proteins are alkylated with urea present, followed by successive buffer exchanges. Proteins are digested with surfactants present, then liberated with centrifugation. Extraction with organic solvent leaves behind pure peptides for LC–MS analysis. This procedure reduces losses about 300-fold. Figure adapted from Erde *et al.* (2014) [104].

FASP adapts well to the microscale due to the increase in local concentration of protein in relation to sample volume. Because the filter traps the proteins, it artificially increases the concentration. Recent advances by Erde, *et al.* suggests that the addition of a carrier such as Tween-20 to the molecular-weight cutoff filter before protein addition reduces losses 300-fold [104] (Figure 3). Also, instead of loading urea-solubilized peptides onto the filter unit, 0.2% deoxycholic acid can be used. This technique increases trypsin digestion efficiency, but necessitates cleanup by an additional method; the use of TCA-DOC precipitation post-FASP could be implemented for this use.

### 3.3. Microproteomic Fractionation and Separation

Loss of peptides due to lower peptide extraction efficiency can discourage the use of gel electrophoresis in microproteomic preparations, despite the raw power of the strategy. However, with stringent sample preparation strategies, gel electrophoresis can be used in microproteomics. Cha *et al.* describe the use of SDS-PAGE for the analysis of 60,000 breast cancer cells per specimen, obtained by laser capture microdissection [149]. After lysis with lithium dodecyl sulfate, proteins were separated by 1D SDS-PAGE. The gel lanes were divided into three sections based on molecular weight and subjected to in-gel digestion. This procedure provided three fractions per sample. Eighteen different samples, nine breast tumors and nine normal tissues, were analyzed. Overall, Cha *et al.* identified 2588 unique protein groups from the 18 samples, with only 3% of unique protein identifications, and about 78 protein groups, found in more than one sample. This study illustrates that, while loss is a real concern for in-gel digestion, useful information can be obtained from small sample amounts using 1D gel fractionation. Gel electrophoresis can be an excellent choice for microgram quantities of sample.

Other electrophoresis techniques exist that are compatible with microproteomics. Jorgenson and Lukacs introduced the concept of zone electrophoresis in silica capillaries [150]. Capillary zone electrophoresis (CZE) is perhaps best known for its use in the Human Genome Project [151]; the technique has grown on the fringe of proteomics [152] but is now gaining wider appeal and has been applied to proteomic separations with excellent results. Li *et al.* demonstrated the use of CZE with a sheath-flow interface for analysis of a moderately complex bacterial proteome [153]. This work has been further advanced by Sun *et al.* for more complex proteomes [154] and quantitation by multiple reaction monitoring on a human cell line, the first analysis of its kind [155]. Zhu *et al.* automated the process using a PrinCE autosampler for *Escherichia coli* digests [156]. Most impressively, Zhu *et al.* demonstrated the use of CZE for the analysis of single nanograms of material for proteomic analysis, identifying more peptides than an UPLC–ESI-MS/MS system [157]. The advantages of CZE lie in its speed and sensitivity, as compared to UPLC; a single separation consisted of 50 min of mass spectrometry time for a 60 centimeter capillary. Similar amounts of time were used for UPLC in this case, but most of the peptides for CZE had eluted by ~30 min for each loading amount. CZE showed much better sensitivity, resolving peptides at the 1 ng amount, where the UPLC showed little to no signal above background. The weakness of CZE is its low loading amount, due in part to the small volume of the 50 μm inner diameter (ID) capillary and zero peptide retention on the stationary phase. This analysis shows that CZE is a viable micro- to nano-proteomic separation technique, lowering sample requirements while retaining sensitivity and providing numerous peptide and protein identifications, albeit complementary to UPLC. CZE has also been applied to top-down proteomics as demonstrated by Li *et al.* [158] and Zhao *et al.* [159].

### 3.4. One Application of Microproteomics: Exploring Cancer Samples

Cancer is one of the leading causes of death in the United States, and the growth and developmental mechanisms of tumors are poorly understood [160]. Tumors have substantial cellular heterogeneity, making tumor biology a critical area of study. Microproteomic techniques are valuable tools to explore the complex proteomic differences within a single tumor [161]. Many of the approaches used in this review are techniques that can be applied to tumor analysis. Indeed, tumor biology and biomarker discovery are major driving forces for proteomic analysis, based on the number of publications on proteomic cancer analysis in recent years.

Proteomics is useful for tumor biology due to the breadth of protein information obtainable. Often, diagnosis and evaluation of tumors are done with histology and immunohistochemical analysis [162]. Biopsies are sliced, stained, and analyzed with microscopy. While accurate, precise, and mature, the throughput of this technique is relatively low. Immunostaining and histochemical staining are commonly used for cancer diagnosis, but they are limited techniques. Only one or two proteins can be visualized with traditional microscopy techniques. Tumors can also be biopsied using needle core or aspiration biopsies. The typical diameter of a needle core biopsy is about 1 mm across [163] and about the length of a grain of rice [164], limiting sample amount and thus the breadth of analyses that may be possible. However, microproteomics can bridge this gap, providing more methods for tumor analysis to complement mature techniques. Thanks to laser capture microdissection, both histology and mass spectrometry can be performed on the same sample. Diagnosis, proteome analysis, and network analysis can be consolidated, and microproteomics can help ensure maximum data from minimal material. Meanwhile, thousands of protein groups can be identified in a single mass spectrometry run. That information is then uploaded into network analysis databases, providing an in-depth look at a tumor’s molecular equilibria. Mass spectrometry-based strategies can identify targets for cancer therapy. Analysis of pathways in cancer can not only give scientists and medical personnel insight into the workings of cancer; it can also give more immediate treatment options, possibly ruling out ineffective therapies or encouraging more productive, less deleterious chemotherapies.

Model systems for cancer can be used in place of primary tumor samples, which are precious biological samples. Two-dimensional (2D) cell culture and murine model are both useful model systems with unique advantages and disadvantages. 2D cell culture is least likely to be used for microproteomic analysis; great quantities of cells can be cultured in a single flask, resulting in milligram amounts of protein available. Furthermore, clonal lines can be grown in parallel and combined. For cancer researchers, this is an advantage. 2D cell culture is a high-throughput, relatively low-cost technique, at the expense of the model’s accuracy. Murine models, while expensive, have more accurate representations of human tumors. Tumors obtained from mouse models are reproducible, yet unique to the individual organism. The overall size of tumors are smaller, providing less protein; coupled with other analyses, microproteomics is a likely candidate with the sample available. Further, even without direct tumor analysis, inferences and data can be obtained using tests similar to human techniques, such as blood and plasma [165,166]. 3D cell cultures recapitulate the tumor microenvironment to a high degree, but also retain many of the advantages of 2D cell culture. Deriving from clonal cell lines, 3D cultures (“spheroids”) are reproducible in their growth patterns, but display intraculture chemical and cellular heterogeneity. Most often, 3D cultures are analyzed in bulk. We have found that, in certain cell lines, these cell cultures consistently provide around 40 μg of protein in a 1 mm HCT116 spheroid [167]. Analysis of single cultures under various conditions using microproteomic techniques is a viable next step in multicellular spheroid characterization.

## 4. Conclusions

The field of proteomics continues to present solutions to unique challenges of small sample analysis. With advances in technology and methodological breakthroughs, microproteomics pushes the boundaries of analysis down to the biological dynamic range. Progress into the lower reaches of biological activity requires greater reproducibility and specificity of techniques, to analyze specific subsets of tissue with greater confidence. Increases in instrument sensitivity and more compatible preparation techniques are necessary advancements for robust analysis of micro- and nano-scale samples, especially in the analysis of membrane proteins.

The future holds many options for the field of microproteomics. The development of methods for efficient removal of contaminants, such as FASP, provides paths to analyze single, fixed mammalian cells with minimal loss. While obligate losses exist, the analysis of single-cell proteomes remains a major goal. Microdissection using lasers has already miniaturized the dimensions of sample collection; separation techniques for UPLC or CZE need to follow this miniaturized approach to provide new workhorses with minimal sample dilution. Robotics may replace hand-held instruments to provide precise handling of specimens and samples, increasing speed of analysis and reproducibility of results. The field of microproteomics can build on the solid foundation already laid, and with some inventiveness and visionary techniques, the limitations of current techniques can be surmounted.
